# Older Autistic People, Access and Experiences of Services, and the Factors That Affect This

**DOI:** 10.3390/ijerph21111535

**Published:** 2024-11-19

**Authors:** Marion Hersh, Panda Mery, Michael Dawson

**Affiliations:** Biomedical Engineering, University of Glasgow, Glasgow G12 8LT, Scotland, UK; panda@forestlim.com (P.M.); michael.dawson@glasgow.ac.uk (M.D.)

**Keywords:** older, autistic, services, strategies, access, sensory, communication

## Abstract

This paper presents new empirical data obtained from interviews and focus groups on older (50 and over) autistic people’s experiences of accessing a variety of services. The involvement of older autistic people and giving voice to their experiences was central to all aspects of the research process. This work makes a significant contribution to the scarce literature on older autistic people. In particular, it discusses the factors that act as barriers and enablers to the access to and productive use of services, the strategies used by participants to manage and improve their experiences, and the success of these strategies. It shows older autistic people as autonomous adults and active protagonists in their own lives, taking action to overcome the barriers they experience to accessing services on the same terms as everyone else, but that lack of understanding and support from service providers and the general public can undermine their strategies. Finally, this work provides a series of recommendations for service providers to improve (older) autistic people’s service access and experiences.

## 1. Introduction

Autism research and service provision has focused on autistic children, with, more recently, some attention being paid to (younger) adults. Research and service provision for older people has generally ignored older autistic people, including their particular sensory, communication, and other needs. Despite the number of older autistic people in the population, accurate data on this figure and how it is changing over time are not available. Calculations based on prevalence rate and population estimates [1,2] give estimates of between 2.96 and 7.63 million autistic people aged 50 and over in the European Union. The wide range indicates the need for further research to obtain more accurate data. It also illustrates the lack of attention to this group more generally, including in research and service provision. We have chosen to use the lower age limit of 50 in our definition of ‘older’ due to its use in several previous studies, e.g., [3,4], and its use by our funders, who use it in their definition of ‘older’.

The lack of research on older autistic people has been noted [5]. The limited research to date has tended to focus on their mental and physical health, e.g., [6,7,8], and, to some extent, also their quality of life, e.g., [9]. Many of the studies that include older autistic people cover a wider age range than 50 and above, and this age group generally has low representation within them [10].

There has been limited research on the lives of older autistic people and their experiences, desires, and aspirations, the barriers they encounter and the strategies they use to overcome these barriers. There are data on the low educational and employment rates of autistic people, e.g., [11,12], but there is a lack of studies on the experiences of older autistic people in the workplace and the support they require in order to enter or remain in employment. There is some, but limited data on older autistic people’s use of technology, which has shown them to be frequent users of modern information and communication technologies [13], with limited information beyond about age 64.

Most of the research has been carried out by neurotypical people and there has been a focus on changing autistic people’s deficits [14] rather than the social, attitudinal, and infrastructural barriers that exclude them. The importance of involving autistic people in research about them is increasingly being recognised. There have been a number of initiatives. They include the UK Participatory Autism Research Collective [15], which aims to promote autistic involvement in research, has organised a number of conferences and workshops, and was involved in the publication of the Neurodiversity Reader [16] and the US Academic Autism Spectrum Partnership in Research and Education [17], which carries out community-based participatory research to improve the lives of autistic people and has, for instance, published work on autistic burnout [18]. However, there is more limited work on older autistic people being carried out by older autistic people.

This paper will focus on older autistic people’s access to and experiences of service use. This is an important topic in the area of participation in society and will contribute to filling many of the gaps identified in this introduction. In particular, we will be using data from focus groups and interviews with older autistic people to answer the following two research questions:

1. What factors affect older autistic people’s access to and experiences of using services?
2. What strategies do older autistic people use to manage and improve this access and these experiences and how successful are they?

This paper is laid out as follows. A literature review is presented in Section 2 and the materials and methods in Section 3. Results are presented in Section 4 and discussion and responses to research questions in Section 5. The paper is finalised by conclusions, including a list of recommendations for service providers, in Section 6.

## 2. Literature Review

There is a lack of literature on service provision and the experiences of service use of older autistic people. The available literature on autistic adults has tended to focus on the details of services and outcomes or the percentages of people using particular services rather than the experiences of autistic people. Therefore, this section will cover the literature on experiences of service use by autistic adults of all ages, and some studies which also include children, as well as studies covering suggestions for improvements. However, it will not cover studies of service use that purely provide (comparative) statistical data on service use and/or unmet need and do not cover user experiences.

Sensory issues have been found to have a significant impact on experiences of service use. Maclennan et al. [19] identified six factors that made public spaces ‘enabling’ or ‘disabling’ in sensory terms, but the majority of their participants (*n* = 24, age 18–44) were under 35. These factors were the nature of the sensory input and its duration; whether it could be avoided and participants had control over it; the nature of the space, including how busy/crowded and closed in it was; predictability including whether advance information was available; the extent of understanding by staff and members of the public; and whether spaces to recover in, such as a quiet room, were available. Supermarkets; eating places, e.g., restaurants, high streets, and city/town centres; public transport; healthcare, e.g., doctors’ surgeries and hospitals; and shopping centres and retail shops, with some exceptions such as book stores, were found to be particularly problematical. Outdoor spaces, museums, and various performance venues were considered better in sensory terms. Older autistic people (*n* = 15, age 50+) have been found to use noise-cancelling headphones and play music to try to manage sensory issues, as well as avoiding or minimising exposure [20].

The available communication options for contacting services and their suitability for autistic people have also been found to affect (older) autistic people’s ability to access services and their experiences of using them. Two studies including older autistic people (*n* = 223, *n* = 43 for age 51–74) [13] and (*n* = 15, age 50 and over) [20] found that (older) autistic people frequently preferred asynchronous written communication such as email and instant messaging. Telephone use for contacting service providers caused difficulties to many (older) autistic people, but the option to use email or online forms was frequently not available or had slow response rates [13]. They also preferred email and found telephone-only communication a barrier to communicating with unfamiliar people [21]. Autistic people have been found to use video calling for work, study, and social interaction and to experience stress when using it due to bright lights and background noise, cognitive overload, worry about technology problems, and missing social cues. They managed this by connecting with the video off, minimising bright lights, adjusting the volume, taking notes, and faking eye contact [22].

There is a small body of literature on autistic people’s negative experiences with and difficulties in accessing health services and suggestions for improvements. Particular problems resulted from providers’ lack of knowledge of autism, lack of consideration of autistic people’s sensory and communication needs, and negative attitudes and stigma towards autistic people [9]. Doherty et al. [23] (*n* = 507, age 17–73, 157 controls) found that nearly 80% of autistic participants found visiting a GP difficult, leading to delays in accessing treatment (60%), requiring more extensive treatment or surgery (36%), and untreated serious conditions (34%). Autistic people have been found to have greater ‘unmet health needs’, to make greater use of emergency services and less of preventative ones than non-autistic people, and to experience worse communication with professionals and lower patient self-efficacy [24]. The Westminster Commission on Autism [25] found that the overwhelming majority of autistic people do not think health professionals understand their needs as autistic people and are concerned about sensory overstimulation, empowerment, and the attitudes and lack of knowledge of healthcare professionals.

Communication issues discussed in the context of health service use include whether written communication is permitted and accessible language used, and difficulties describing symptoms [9]; the use of non-verbal communication, slow processing speed, and difficulties in understanding people [26,27]; as well as unhelpful non-literal interpretations, social communication needs and emotional processing differences [27]. The sensory issues affecting autistic adults’ healthcare experiences include body awareness, particularly the experience of pain [26]. Healthcare personnel have been found to require training in all aspects of caring for autistic adults with particular stress on communication and identifying and making accommodations [28]. This includes 40% of GPs lacking autism training and having limited confidence in working with autistic patients [29].

Autistic people have also been found to be concerned about healthcare professionals’ lack of knowledge of autistic people and the need for respectful attitudes and treatment, and appropriate skills [26,27]. This lack of knowledge has impacted autistics in need of healthcare, as no accommodations or tailored information were provided to meet their needs, including sensory ones. In addition, the frequently different ways in which autistics experience pain are not understood. And, their health concerns are dismissed as just a part of autism [25].

A survey of the diagnosis experiences of 128 so-called ‘high functioning’ autistic people [30] found that just under half (47%) were very or quite satisfied and about two fifths of respondents were ‘very’ or ‘quite’ dissatisfied. The majority were satisfied with the information received and how it was provided, but under a quarter with the support received [30].

Suggestions for improving autistic adults’ access to and experience of healthcare services cover the healthcare environment, appointments, communication, and taking account of sensory issues and body awareness in physical examinations [23,24,31,32]. The healthcare environment should be ‘autism friendly’ with quiet waiting rooms, a lack of clutter, and taking account of sensory issues of all types. Autistic people should have the option for the first or last appointment. Communication recommendations include the option to email the consultation reason in advance, text or online appointment booking, allowing the use of preferred forms of communication such as writing or typing, using precise specific language and providing very detailed step-by-step written instructions for obtaining prescriptions, tests, and referrals, allowing extra time to process information, and clarifying the role of support personnel. Health professionals should have training in communicating with autistic people. Suggestions for improving counselling services include therapists working ‘collaboratively and respectfully’ and the provision of clear information and opportunities for discussion, for instance, of the reasons for the therapeutic approach chosen and what might be helpful [27]. Other suggestions included annual health checks [25] and a more structured diagnostic process [30].

An Autism Healthcare Accommodations Tool has been developed by the Academic Autistic Spectrum Partnership in Research and Education (AASPIRE) and found to be useful in preparing for visits and helping autistic people communicate their medical needs in a survey of 259 autistic adults and 51 healthcare providers [33]. The Checklist for Autism-Friendly Environments has been developed to support healthcare practitioners make the healthcare environment more accessible for autistic patients [32].

Access to transport has been found to be another area causing difficulties to autistic people. Autistic adults (*n* = 295, mean age 40.8 years, 146 controls) have been found to have less access to public transport or a private car and to be less likely to use either [34]. This disadvantages them in terms of education and employment, as public transport use was found to lead to better educational outcomes, and public transport and car use to improvements in both educational and employment outcomes. The difficulties in using public transport and cars are further borne out by two US studies, which found that autistic people (*n* = 22, age 18–64, 19 parents of autistic adults aged 18–49) and (*n* = 703, age 18+, mainly completed by others on their behalf) most commonly travelled as a car passenger with the associated disadvantages of dependence on others [35,36]. About 40% of the participants in the second survey experienced difficulties getting to stops and stations and were concerned about treatment by other passengers and drivers, and about half of the routes they needed were not covered. Further evidence of the link between employment and access to travel comes from a US survey (*n* = 486 employed, 634 unemployed, mean age 28, standard deviation 9.8), which found that employed participants experienced fewer barriers to public transport use and were more likely to drive themselves than unemployed ones, who were more likely to travel as car passengers [37].

## 3. Materials and Methods

### 3.1. Research Questions, Protocols, and Recruitment

The data presented in this paper will be used to answer the following two research questions:

1. What factors affect older autistic people’s access to and experiences of using services?
2. What strategies do older autistic people use to manage and improve this access and these experiences and how successful are they?

This research was carried out by a team of three older autistic people: an academic researcher (M.H.) and two older autistic peer researchers (M.D. and P.M.). A survey of older autistic people was carried out prior to the work reported in this paper. It covered their living and employment situations and use of technology, and the tools and support that would be useful to them and the types of information to which they would like signposting. As a team of older autistic researchers, it was important to us to hear directly from older autistic people and involve older autistic people directly in the research. In this research, the team was supported by an Advisory Board comprising three older autistic people, but health and other personal circumstances meant they were unable to continue their support for the research reported here.

The first draft of the questionnaire was produced by the research team. It was then provided online to the Advisory Board for comments and discussed on Zoom. A revised version was again made available online for further comments and the resulting revised version was piloted online with five people. A password was used during testing with the Advisory Board and piloting to restrict access to only these groups and removed before the questionnaire was made publicly available.

As a result of the advice from the Advisory Board and piloting, the questionnaire was significantly modified. In particular, most of the questions about the type of technological support and features of an app or website that would be useful were rewritten to avoid difficulties in interpreting them and to make it easier for participants to respond to them, and many of the closed questions were replaced by open ones. The wording of a few questions was modified and some additional response options were added, including the option ‘Guide to autistic-friendly spaces’ to the question ‘Which of the following tools and support are of interest to you?’ Therefore, the co-production process resulted in a questionnaire that was much easier for participants to answer. Since the questionnaire could be considered the foundation of all the research, this resulted in a much better basis for it than would have otherwise been the case.

Ethical approval for both the survey and the follow-up interviews and focus groups was obtained from the Ethics Committee of the College of Science and Engineering of the University of Glasgow.

This survey included multiple choice questions about the tools and support that would be useful to participants and the types of information to which they would like signposting. Statistical analysis of the results identified that participants’ greatest interest was in information about autistic-friendly spaces and services and support for executive functioning. Follow-up focus groups and interviews were organised to obtain more detailed information and draw up specifications for the autistic-friendly spaces and services and executive functioning systems. Identical questions were used in the interviews and focus groups. Two series of questions with follow-up prompts were drawn up, one for each of the topics. The initial drafts were produced by M.H. and revised through discussion by the whole team to produce the final versions. The questions are presented in Appendix A.

The main approach to recruitment was through follow-up contact details of questionnaire participants who had agreed they could be contacted for this and provided an email address. They were emailed with the focus group information sheet by M.D. Some of these participants responded that they would prefer to participate individually rather than in a focus group. Their details were passed on to M.H. who emailed them with the interview information sheet. In both cases, positive responses were followed by further emails to arrange times and dates.

Each of the two topics was the subject of a separate focus group, whereas they were discussed sequentially in the interviews. Therefore, M.D. also asked participants which focus group(s) they wanted to participate in. In addition, one of the focus group participants asked if they could bring an older autistic friend to the focus group they were participating in and this was allowed. All focus group and interview participants were sent the list of questions without the prompts in advance, though one interview participant was unintentionally sent the version including the prompts.

Due to continuing lockdown, all the focus groups and interviews took place at a distance. Therefore, electronic versions of the information sheets and consent forms were sent to the participants in advance for their signature. The focus groups took place on Zoom, whereas interview participants were given the choice of a phone or written option. The lists of questions were screen-shared in the focus groups with the main question being presented first and the prompts subsequently to encourage further discussion. All three researchers participated in the focus groups, whereas the interviews were carried out by M.H. The focus groups and interviews were all recorded on a digital recorder and transcribed verbatim.

### 3.2. Analysis

After discussions following the interviews and focus groups, the team agreed that the initial focus would be on autistic-friendly spaces and that further work and funding would be required to develop a system to support executive functioning. Two stages of analysis of the results of the interviews and focus groups were carried out based on the transcriptions. The first was a high-level analysis to obtain the specifications for the autistic-friendly spaces system, with P.M. taking the lead and drawing up the first set of specifications. This was followed by discussion and modification of these specifications to produce updated versions. Once we all agreed on the specifications, we sent them to the software firm we were working with, Reborn Media, and received a request to prioritise them. P.M. produce a revised version dividing the specifications into three priority levels. This was again followed by discussion and modification. The final version was sent to Reborn Media to develop a website for older autistic people to provide reviews of autistic-friendly spaces and services and, subsequently, a forum for older autistic people to support community building. The choice of a website was in line with participant preferences expressed in the survey. The second stage of analysis was thematic analysis [38], with M.H. taking the lead. Since this paper discusses the results of the second-stage thematic analysis, we will not discuss the methodology of the first-stage analysis further.

The first step in the thematic analysis was the identification of first-order themes without a reference framework to obtain an overall understanding of the data. This involved the inductive coding of each element of each focus group and interview. The next step involved identifying and combining related codes and tabulating the resulting codes for each focus group and interview. This was followed by determining the main themes represented in each or the majority of the interviews and focus groups and splicing to produce a small number of themes which encapsulated these themes. Further analysis involved extracting data relevant to each theme from each interview and focus group and grouping it together.

### 3.3. Participant Overview

There were three focus groups with three participants in each one. Two participants attended two focus groups, one on each topic. The other five participants each attended one focus group, giving a total of seven different participants involved in the focus groups. Four people participated in interviews. There was no overlap between interview and focus group participants so that 11 participants in total were involved in the focus groups and interviews.

We did not collect demographic data from participants at the time of the interviews and focus groups, and the early anonymisation of the data means that we cannot access the demographic data subset for just these participants. We are, therefore, unfortunately unable to present demographic data for the participants. However, we do have information on the people who provided contact details and can, for instance, deduce from this that, unfortunately, there were no Black and ethnic minority participants or participants with learning disabilities in the interviews and focus groups.

## 4. Results

This section is divided into three main subsections. The first two cover the answer to the first research question on the factors that affect experiences of service use. The second subsection covers positive experiences and the factors that affect this. The results related to the second research question on the strategies used are presented in the third subsection.

In this and subsequent sections, interview participants will be referred to by anonymised initials and focus groups participants by FG followed by a number.

### 4.1. Negative Experiences of Service Use and the Factors Responsible

This subsection is divided further into four subsections based on factors that lead to negative experiences: sensory issues, communication issues, the impact of ageing, and bullying, harassment, and other issues.

#### 4.1.1. Sensory Issues

Sensory issues were the most frequent cause of negative experiences of service use and were mentioned in all the interviews and focus groups. Noise was the most common ‘culprit’, including in hospitals, airports, open offices, public transport, restaurants, bars, and schools. The narrative here is of older autistic people trying a variety of spaces and services and experiencing various problems with them, largely on account of noise, but also due to unsuitable lighting and smells. Particular subthemes included sensory issues in spaces intended for autistic people, specific sources of problems, pain, anxiety, and disruption, and lighting and smells.

KE experienced difficulties in finding spaces and services designed specifically for autistic people, and even these were not totally suitable. For instance, a peer support group was in ‘quite a small room so everybody had to … squeeze in together so it felt uncomfortable. I didn’t feel like I had a lot of personal space’. Socialising in the local autism hub was also difficult. ‘In the actual bar … it was very noisy. There was music on … and … it was really echoey. … It was very tiring to talk and concentrate for longer periods of time. It wasn’t a good place to socialise’.

Specific sources of noise that could make service use difficult included hard floors and multi-layer alarms. FG2 was disturbed by the noise from hard floors, which also interfered with them hearing conversations close to them. ‘All the restaurants, the cafés, everywhere has hard floors. … I can hear people talking a couple of tables away … their entire conversations, but I can’t actually hear someone talking right next to me because my background and foreground are combined’. FG2 experienced difficulties due to multi-layer fire alarms. ‘There is a fashion for multi-layering currently, they put in a voice command … a base note alarm and … a top soprano really awful one … choose one and warn people what it sounds like, and give assistance to people who immediately will not like the sound and will either drop to the floor, hide’.

Noise, including that associated with service use, could cause pain, depression, anxiety, and major disruption to those nearby. For instance, for FG2, ‘if somebody drops something … near me on a hard floor, I get like an explosion of pain inside my head. … we’re absolutely convinced … one of the reasons that causes me depression is because … if it goes to the pain centre of my brain I’m in pain the whole time’. Their living situation was also becoming untenable due to transport noise levels. ‘We’re actually going to have to move because I keep getting severe depression, and I think part of that is … it’s extremely noisy where I live … there are two airports and the mainline train’. DN was also being negatively affected by sensory overload, particularly when they had to wait to access services. They had experienced ‘a lot of anxiety and I didn’t realise that much of that was to do with a sense of sensory overwhelm, managing things in terms of executive functioning … which … leads to anxiety’. Waiting was a particular problem. ‘The longer I’m waiting, the more overwhelmed I’m feeling by the sensory overload around me… and I struggle … with having to wait generally, having ADHD’.

Lighting and smells could also cause problems. FG4 commented on this and the wider community affected by sensory issues. ‘Noise and lights, artificial lights, are really, really bad … for me, and … smells … I also have fibromyalgia … Bright lights, … artificial lights, noise, smells … are really, really bad for fibromyalgia as well. … So I think it’s actually probably a lot more common, as issues or as things that are triggering people than just the autistic community. … it’s a shame that it seems so difficult sometimes to get the message through’. Lighting and smells on public transport made it difficult for FG6 to go anywhere. ‘Would it be that hard to have a few buses that were quiet buses, for people that don’t like noise, like me. But then, you’ve got the smells. … I’m really hypersensitive to a lot of things, so I’m a bit of a hermit, nowadays’.

#### 4.1.2. Communication Issues

The mismatch between participants’ communication needs and the communications provided by service providers was another major theme and barrier to service use for participants. Subthemes included written communication, precise instructions, and avoiding unstated assumptions. Participants generally wanted the option to communicate in writing with a named person they could email and not be forced to communicate with services by phone. They needed very precise, clearly defined, and unambiguous information and an avoidance of unstated assumptions, and found non-verbal communication a barrier to comprehension.

FG4 needed very precise information without any ambiguities to avoid confusion. They were ‘extremely specific and detailed. So if something is said which can be interpreted … my brain is giving me both ways and I will be so confused. … I always have plenty of different ideas about interpreting things. **…** I don’t have a clue what people are saying … I tell them how I can see it, and they’re looking at me as if I were from another planet. … And I’m thinking if you say it this way without being more specific, I don’t understand. … If you just add one more word, or whatever else to qualify what you’re saying, then suddenly I know exactly what you’re saying’. Their focus on details was helpful in their career, but interfered with general communication. ‘I would pick on every single detail. … That was really helpful in my career, and … to manage people. Because they knew what I wanted, and how I wanted it. … In real life, that’s a problem, because … people think, you just go on, and on, and on, which I really don’t think I do, or … why do you say that, it’s obvious’.

Communication problems were a particular issue in the healthcare system. RD experienced problems in hospital appointments due to imprecise questions and the lack of information in advance which provided context for the questions. ‘Even the consultant today was asking questions like is your health generally good? … Is there an objective standard that I’m supposed to measure myself against? … Reading the leaflets that’s when the questions made sense. … if I’d have had even a sense of … those questions … in advance … I could have thought about it and … given answers that were more accurate’. Conflicting information related to hospital appointments also caused them problems. ‘The GP said … they’ll send you a letter … the letter arrived and it said ring this number to book your appointment, and I rang the number and they said, oh, no, no … we will send you a letter telling you when your appointment is. So … I followed the rules and it wasn’t right, and that’s really stressful. … The guidance I was sent … said you’re really welcome to bring somebody with you … and I thought great I can take my husband … There was also a COVID sheet … please don’t take anybody with you unless you really need to … So partly it’s about not knowing the rules, and partly it’s about not knowing what it’s going to be like, because there’s always the possibility of an unexpected sensory overload’.

DN had experienced difficulties with the need to phone to obtain hospital appointments. ‘If … you must phone up for an appointment and it’s a switchboard number and you’re stuck in a queue … I’m really not going to be phoning them unless … I feel I have no choice and I find it exceedingly stressful. … Particularly, since the pandemic … getting through to people is really difficult. … not … getting equal access to medical provision as everyone else’.

Imprecise instructions were a source of problems for several participants, particularly in the case of directions. For instance, FG1 found that ‘people look like you’re stupid because you can’t follow a set of instructions given by someone that clearly knows where they’re going but can’t translate that into the experience of someone who doesn’t … that starts to compound with … being overstimulated and it makes the entire thing so difficult … that by the time you get to where you’re meant to be you’re so overwrought that you can’t … do anything in the appointment’. FG2 had problems with hospital signage. ‘My big bugbear is hospitals. … I can’t follow the signs at all. I finish up asking loads of people directions, and then I can’t follow their directions either’.

The use of non-verbal communication could act as a barrier to understanding. For instance, FG2 could not ‘follow instructions because half … are in non-verbal cues and I cannot read them … non-verbal communication … and you’re autistic and can’t read it, then you miss a fairly large proportion of what they’re trying to communicate to you’. Being approached too often could also be a problem. FG3 considered ‘one of the things is instead of being approachable … people approach you too much. Like if you’re in a restaurant … and you get the waiter coming over every five minutes to ask how the food is. I just want to eat my food’.

#### 4.1.3. The Impact of Ageing

Several participants experienced changes over time which could affect their ability to access services and their experiences of using them. RD considered ‘I’m definitely not as resilient as I used to be. I used to run … very close to burnout. … I just can’t do that anymore. … I get more sensory distress and more sensory overwhelm. … I’m just not recovering the same way anymore, so I just have to be a lot more careful with things like noise. … When I was younger I could just have a nap for 20 min and reset. Now that’s more like an hour, hour and a half, and you can’t do that all the time’.

Participants’ needs and ability to find alternatives to not very accessible services could change over time. For instance, RD was using a combination of a private car and public transport, and found their car much easier to use and worried about the time when they would no longer be able to drive. ‘I think transport is a real problem. I can still drive relatively successfully. I’m not sure whether I’ll be driving when I’m 70 … then … you have to be taking public transport and that feels exhausting … my car doesn’t, because it’s my car and it’s comfortable and it’s predictable’.

FG2 had less ability to screen out noise compared to when they were younger. ‘And when I was younger I used to screen it out. I always knew I lived in a much louder world than everybody else and … I could hear things that no one else could, … but I didn’t know I was autistic until really recently. … As I get older I’m less and less and less able to counter the sound sensitivity, so I have to wear ear defenders everywhere, including supermarkets’.

FW was finding it more difficult to deal with previously appropriate spaces over time. ‘For the past two years I’ve found it difficult to deal with spaces which years ago I found congenial for autistic people. … years ago when we would go into … archives, libraries and/or offices … I forced myself to get there … and do the work. … But I increasingly find it more and more difficult to cope. … more and more difficult … distractions. My concentration isn’t as good as it was. … I’ve become even more inflexible now in some ways than I was years ago. I think that’s where life has worn me down’. They also found it ‘more difficult with the heat … as the climate is getting warmer in summer … the heat actually makes me so ill literally I cannot function properly’.

FW was also concerned about the lack of appropriate assistance. ‘With the railways the proposal to abolish all the ticket offices … fills me with horror because using machines is absolutely beyond me, especially if … there’s a problem. … you may have someone on the gate at the station that knows the job. … if it’s purely automatically if there’s a problem you just keep away. … In many ways … society has become … easier for people like myself than it was 30, 40, 50 years ago. But in many ways it’s much, much worse. … when it was the human touch’.

#### 4.1.4. Bullying, Harassment, and Other Negative Experiences

The issues discussed in this section include bullying and harassment by other travellers, the lack of post-diagnosis support, and unsuccessful attempts to adapt services and events. FG4 had had some very distressing experiences of bullying and harassment when travelling by bus. ‘I can hardly walk or stand up, and a lot of the time … when I ask for a seat … I have to justify it … to get my freedom pass out … to say, “I’m sorry, I’m disabled, I cannot stand”. And then they say, “why can’t you sit somewhere else”, and I say, “because unfortunately I need this bar to get up and down, and if I go somewhere else … I’m not going to be able to safely sit or stand”. … At times it gets me in tears. … they were just winding other people up, loudly, about how demanding, and horrible I was … I just left, and I just collapsed, literally, I just sat on a bench in the common … and I just was in tears, for such a long time. Because I felt so belittled, … so undermined, and … assaulted. … This happened more than once. … if there’s no space on the Tube, and I’ll ask for a disabled seat … people tell me, “but you’re too young to be disabled”. … That’s kind of really irrelevant, it’s not a question of age, anyway, and it doesn’t necessarily show. … Or, “you don’t look disabled”—well, no, perhaps not, if I stand up for half a second … or … I’m warmed up’.

DN had found it very difficult to obtain post-diagnosis support due to a combination of difficulties in accessing information and a lack of funding. ‘There was no post-diagnosis support. … trying to find things out as I’ve gone along. … I wasn’t aware of any autism friendly networks … it’s taken a while to come across those. One of the first ones … came on the TV. … I checked the website, and … realised that there were autistic advocates within my local area. I wasn’t able really to use their services … because … I didn’t have access to funding in any form really’.

Sometimes attempts to make events accessible were unsuccessful and could lead to distress when the wider impacts of particular measures were not considered. For instance, KE ‘was actually looking forward to seeing my colleagues because I haven’t seen them face-to-face for a long time but the COVID spaces were, right at the back and excluded … from everybody else. … I ended up having an autistic meltdown … I couldn’t stop crying’. Colleagues and managers who wanted to be helpful but did not understand the issues could also be mystified as to why an autistic person was becoming upset. ‘One of the managers was very nice but made me sit with her until I calmed down. I couldn’t really explain to her that I just wanted to be left alone so I had to mask and force myself to look like I’d calmed down so that I could go. I think that they want to support me but … they’re not really sure how’.

### 4.2. Positive Experiences of Service Use

Some participants had positive experiences of service use. Subthemes included positive attitudes and greater understanding, autistic-friendly approaches to sensory or communication issues, accessing services, including online without human interaction, and appropriate assistance. KE had a good experience ‘in the hairdressers when I said I was autistic and she made it comfortable for me, she said her own son is on the spectrum. So I think that helps because she had a little bit of understanding‘.

DN found a local service which was ‘very, very friendly … and very helpful, really. When funding became available … they offered me a place on a late diagnosis group … with a small number of other recently diagnosed autistic adults and they have also provided some funding for counselling from a counsellor who is also autistic, so understands the challenges that autistic adults can face. … if I’ve had specific issues … they … have tried to help in whatever way they’re able to. They also have been running webinars on a variety of themes relating to autism and these have been very useful as well’.

FG5 recommended an ‘art and technology centre’ which was well designed in sensory terms. ‘And they actually designed the space to be autistic-friendly … right down to details like … all the cups in the cafe have cork mats underneath them, so that they don’t make a noise when you put them down. … All the walls are in kind of low arousal colours … And there’s obviously a lot of understanding of autistic issues in that environment’.

FW generally liked the sensory aspects and comfort of libraries, as well as the possibility of assistance. ‘Libraries … are usually quiet, comfortable, well-upholstered, simple, well-lit. Suitable cognisant support, when to approach, human touch, understanding. I do find natural light helpful … I do find it helpful to have more of a cosy atmosphere’.

RD liked their optometrist, as they were respectful, understanding, and allowed more time for processing, and the place was quiet. ‘I’m under the care of a behavioural optometrist at the moment … and I was really delighted in how autistic-friendly the process was … it was really clear to me why and how. … He’s upstairs … there’s no extraneous noises, it’s relatively calm, but … there’s fun things to look at as well which I think is really helpful. More than anything … he understood I needed time to process … no optometrist has ever done that for me before. … He actually paused between them [putting lenses in front of RD’s eyes] and just that pause of a few seconds was enough for me to be able to … figure out which one works now. … I really was aware of my brain needing the time to process. … He was welcoming of my differences without seeing them as childish’.

The option to have access to services without personal interaction was appreciated, including through the use of online services. FG3 preferred direct access to services without having to go past a ‘too friendly’ receptionist. ‘The gym I go to now is not really staffed by anyone on a desk, you can just let yourself in … let yourself out. … You really don’t have to talk to anybody at all. And that … is a lot friendlier than having to walk past a desk where someone’s … saying, “hello, hi, what are you in for today”. I just want to be left alone to work out’.

RD generally preferred online services. ‘I would always generally use things online rather than not. … If we can possibly order things without talking to a human being online we will do that. … I find online services often quite good. The one exception … is the doctor online service, and it’s because it’s really badly designed. … Just not working for stupid reasons. … This form that one of my employers needed me to fill in … just didn’t work on my browser on my PC … it hasn’t taken account of the possibility that you might be working for more than one institution’.

Although involvement with hospitals and the healthcare system more generally was problematical, participants did have some good experiences. FG1’s local hospital had ‘an autism specialist nurse’. ‘I wasn’t allowed anybody in because of the pandemic, so they sent an assistant to stand with me to go to my appointments … to help me navigate and to come out of the building, and that was very useful’. DN was provided a quiet room to wait in. ‘When I went and presented myself to the receptionist, they said, oh, we’ve got a quiet area for you and they showed me to a private consultation room and then the consultant came and took me to her consultation room‘.

### 4.3. Strategies for Overcoming Barriers

Many of the strategies used by participants involved managing sensory issues, particularly noise. They included the use of protection, asking for music to be turned off and quiet spaces to escape to. Protection was particularly used against noise. For instance, FG2 ‘wear[s] ear defenders all the time’ and ‘everywhere’ and RD has ‘noise reducing earbuds, the loose ones’ and ‘previously … on public transport … I’d worn headphones and played music’. However, KE also used protection against light and would ‘take some ear plugs or some sunglasses’ if they needed to use a service that ‘was very big and noisy and crowded’.

RD had experiences of both successful and unsuccessful attempts to ask for noise to be turned off, with the example of an unsuccessful attempt given first. ‘So in the consultant’s room there was … noise next door that was banging loudly and repeatedly … and I pointed it out, and she said … “it’s terrible, isn’t it?” … I find that so often if I say this is difficult for me that what I get is sympathy rather than … accommodation. I find that very infantilising, because I don’t need the sympathy. … What I need is a change. Or an apology or something suggesting this is going to improve’.

The successful attempt follows. ‘There’s a radio playing in … this tiny room. There’s already two nurses in and they’re going to take eight vials of blood from me, and I did actually say “do you mind if we turn the radio off please”, because there was no reason why it needed to be on. … They did actually look at me surprised and go … “of course we can do that”. And they did do that, because it was an easy thing to change’. RD also commented on the differences in perceptions of autistic and neurotypical people. ‘Things that are minor irritations for neurotypical people aren’t minor irritations for me. That banging … it was loud and it was repetitive’.

FG6 tried to reduce sensory exposure in hospitals by reducing the time they spent inside the building. ‘I always go way before time. … I always try to find how to get from A to B, on the outside of the building, instead of the inside, because of the smells, and because of the people, and because of the lights, and everything else’.

DN tried to acquire the first appointment to avoid waiting and the associated sensory overstimulation. ‘Could I have the first appointment of the day so I’m not waiting too long because I cannot wait. Because the longer I’m waiting, the more overwhelmed I’m feeling by the sensory overload around me … and I struggle … with having to wait generally, having ADHD on top of that’.

A quiet space to retreat to could be useful in preventing or managing feelings of being overwhelmed. FG5 used the airport multi-faith room as a ‘bolt-hole’. ‘Before I was diagnosed, I used to use the multi-faith room in airports, as an autism friendly space. But I’m not religious at all … but every time I had to take a plane, I would always end up in this multi-faith room. … you’d have maybe one or two other people in there, and it would be silent prayer. I could sit and read a book, or something’.

Other people using them to talk could frustrate attempts at quiet spaces. FG6 ‘worked for a local authority … they bought a new building … all this new open office rubbish, that most of us hate, and I was really struggling. … In the end, I managed to have a part of the building, near a window, with a few desks. … Even though it said quiet area, people went there and talked. … No phones allowed, and they would take their … mobiles and talk. And I would politely ask, and I would be met with very aggressive language, and attitudes. … And people would … say, yeah, but we like it here, because it’s quiet. Well, it was before you … made it not quiet. … I always fight hard for everything. So that we can educate people … I also managed to have the quiet room … in the basement. So, whenever I was overwhelmed, I had this traffic light thing, where I would leave the red on my desk … But then, the quiet room became the prayer room. … people used to come to pray, but they made these noises … I only went there when I was really overwhelmed, because of the people talking on the phone. … And in the end, I had to leave. I did get compensation, I hired a solicitor … They had this whole thing that, we are an autistic-friendly local authority. … Things like that beat you down’.

Participants had several calming strategies in addition to using calm spaces. RD had ‘a little furry toy … when you pick it up you startle it … it has a little motor that goes bum, bum, bum, bum, bum, and you stroke it until it calms down and it purrs and eventually it goes to sleep’. FG5 had a device to produce ‘little pinpricks of laser light on the ceiling, and clouds … which I regularly use in my own bedroom, now, as a way of calming me down at night time’ (FG5). These approaches seemed fairly successful.

Participants also had strategies for managing communication. FG3 wanted ‘like a traffic light system of how much conversation you want … so that you can just have a thing to say, please don’t talk to me, because you can always change it if you want’. However, they seemed to be waiting for the system to be ‘rolled out’ rather than implementing it themselves. FG2 tried to use written communication to avoid non-verbal communication and imprecise instructions. ‘I always get people to write stuff down because then they can’t do that [non-verbal communication], and then they have to think clearly when they’re writing it down…. too often when people are giving verbal instructions I’ve got catch phrases I don’t like: over there. … through those doors. It’s so vague. So, I say to them I need prescriptive language: first turn on the left; second turn on the right’.

DN was trying to obtain their GP’s email address, but experienced difficulties in obtaining it. ‘I’ve been trying since October last year to get an email address for my GP so that I can ask for a prescription … and it takes a while for that prescription … to get to the pharmacy for me to know it’s there … and the results of tests’.

Several participants disclosed their autism diagnosis and explained their needs, but success was variable. This generally worked for FG1 and KE at their hairdressers; in KE’s case, this was possibly because the hairdresser had an autistic son. In FG1’s case, ‘I always tell them when I’m trying to book something that I’m autistic and I need a bit longer on the consultation, and I’m likely to bring a picture with me and likely to want it to look something similar to the picture when they finish. And it usually works’. However, FG3 had negative experiences of disclosing their diagnosis to medical professionals, rather than this leading to improved provision. ‘The few times I have disclosed my diagnosis to a medical professional they often look at me disbelievingly or it doesn’t seem to actually make an impact on what they do. I think one person actually scowled at me’.

## 5. Discussion and Answers to the Research Questions

This section is divided into two subsections which discuss the answers to the two research questions. The overall narrative is one of difficulties in accessing services, with negative experiences outweighing positive ones, but participants had a number of strategies to try to overcome barriers and improve their experiences.

### 5.1. Experiences of Service Use

While most of their experiences were negative, participants did also provide examples of (relatively) good experiences of service use. Being understood and having their needs met as an autistic person could lead to better experiences. Participants found that service providers who were themselves autistic or who had autistic relatives were likely to be understanding. Examples included a hairdresser with an autistic son ‘who made it comfortable for me’ (KE) and ‘a very very friendly’ local service which offered DN a place on a ‘late diagnosis group’ and counselling with an autistic counsellor. Other factors which resulted in positive experiences included taking sensory and communication needs into account, including being provided a quiet area to wait in for hospital and other appointments in line with recommendations in Simpson [32], and being able to access the service without the need for personal interaction. RD’s optometrist ticked several boxes. They were respectful, the premises were quiet, and they allowed additional time to respond during testing. FG3’s gym was not staffed and ‘you can just let yourself in’, avoiding the need to interact with a ‘too friendly’ receptionist.

The factors that led to negative experiences were to a large extent the opposite of those resulting in positive experiences. A lack of understanding of or attention to (older) autistic people’s sensory and communication needs had a strong impact on the services they were able to access and their experiences of using them. This combination of communication and noise barriers could leave participants anxious about prospective service use: ‘partly it’s about not knowing the rules and partly … there’s always the possibility of an unexpected sensory overload’ (RD). Sensory overload due to noise was the most common issue, but bright lighting, smells, and crowding were also mentioned as causing problems. Noise could cause pain, depression, and major disruption. For instance, FG2 was ‘going to have to move because I keep getting severe depression and I think part of that is … extremely noisy where I live … two airports and the mainline train’.

The mismatch between participants’ communication needs and the types of communication provided by service providers was another major source of negative experiences of service use. Particular problems resulted from the lack of precise clear information, questions, and instructions, contradictory information, non-verbal communication, the need to phone for appointments, and being ‘approach[ed] too much’, for instance, ‘the waiter coming over every five minutes to ask how the food is’ (FG3). Several of these points parallel the communication issues discussed in the literature in a healthcare context, including whether written communication is permitted, the use of accessible language, and unhelpful non-literal interpretations [9,26,27].

Particular difficulties were experienced on public transport and in the medical system in line with the literature on barriers to accessing healthcare, e.g., [9,23], and public transport [34,35,38]. Unfortunately, these are both important and frequently essential services. This unequal access to health services is likely to be one of the reasons for the poor health outcomes of autistic people, e.g., [9,23]. Access to public transport is also very important for participation in all aspects of modern life, including access to many services, and may be a contributory factor in autistic people’s low rates of employment and education [12,39]. This is borne out in the literature with lower car and public transport use leading to poorer education and employment outcomes [36,38].

Other services where participants experienced problems due to sensory issues included schools, restaurants, bars, airports, and open offices. Even spaces and services intended for autistic people did not necessarily take into account their sensory requirements, such as an autism hub with a bar that was ‘noisy’ and an autism group that met in ‘a small room so everybody had to, kind of, squeeze in together so it felt uncomfortable’ (KE). The types of services in which sensory issues caused problems were largely in line with MacLennan et al.’s [19] types of particularly problematical places from a sensory perspective. The differences may have been due to the different focuses of the research, as well as our participants’ preference for undertaking things online, indicating an avoidance or limited use of, for instance, busy shopping areas.

The focus of most of the comments on sensory issues was sensory overstimulation, particularly from noise, which was experienced as a major stressor which could cause emotional stress, physical pain, or overwhelm, be a contributory factor to depression, and make existing living situations untenable. It could also impact access to healthcare and public transport in line with, e.g., [9,23,38]. However, participants were affected by a wide range of sensory issues and in many different contexts. This is in line with the recommendations in the Checklist for Autism-Friendly Environments to take into account sensory sensitivities including sight, smell, hearing, and body awareness [32]. Participants were also aware that other people’s sensory perceptions were different from theirs and that they were, for instance, disturbed by noise that other people paid minimal attention to. This is in line with sensory issues now being included in the diagnostic criteria for autism spectrum conditions [40]. However, a few participants mentioned both sensory overstimulation and sensory interests, for instance, ‘fun things to look at’ at RD’s optometrist, in line with one of Elwin et al.’s [41] three sensory clusters including sensory interests.

Communication was another major issue for participants. The different communication styles of autistic and neurotypical people could lead to misunderstandings and major stress, and act as a barrier to accessing services, particularly healthcare services, and receiving what they needed from them. Communication problems in healthcare settings and other barriers to accessing services have been relatively extensively discussed in the literature, e.g., [9,23]. Participants wanted other people to use precise clear language for information, questions, and instructions, to write things down and avoid non-verbal signals, ambiguity, hidden meanings, and unstated assumptions.

Particular problems related to incorrect or confusing information about how to make specialist appointments, the need to phone for appointments (paralleling Howard and Sedgwick [21] finding this a barrier), and conflicting information about, for instance, bringing a support person. Participants generally found these situations very stressful.

Difficulties in finding their way around hospital premises combined with sensory issues could leave participants too stressed to benefit fully from appointments, which could again be a factor in autistic people’s poor health outcomes, e.g., [9,23]. Many older autistic people could probably benefit from the option of having someone to guide them to their appointment, as in FG1’s example during COVID-19. The suggestions in [32] for improving signage to make clear the purpose of all rooms would also be helpful.

Several participants found that their ability to deal with spaces and services that were not totally autistic-friendly reduced over time due to factors such as reduced resilience, increased sound sensitivity, and reduced ability to put up with things. For instance, RD was experiencing ‘more sensory overwhelm … and taking longer to recover’ and was concerned as to what would happen when they could no longer drive, as they felt public transport was ‘exhausting’. FW found high temperatures more difficult to deal with and felt they were ‘more inflexible’ than years ago, finding it more difficult to cope with noise and distractions. The literature has generally focused on changes in sensory sensitivity with age for autistic children. The one paper we identified on middle-aged and older adults found a reduction in acuity, but no change in sensory sensitivity [42]. However, increasing age may also have led to a greater ability to accept differences and to find workarounds. These are all important issues where further research will be required.

The lack of understanding and bullying and harassment by the general public could act as barriers to service use, as evidenced by one participant’s very unpleasant experiences on buses when other passengers challenged their need for a seat. This is in line with the evidence of autistic people’s greater risk of bullying and different types of abuse [43,44], and autistic people’s concerns in the literature about their treatment by other passengers and drivers [34,36].

The lack of understanding of what was required and the wider impacts of particular measures could lead to problems even when the intention was to be helpful, as in the case of the provision of ‘Covid spaces’ excluded from everyone else at a work event. KE’s reaction of an ‘autistic meltdown … I could not stop crying’ that was not understood by her manager raises issues of (older) autistic people reacting more strongly to disappointment and difficult situations than neurotypical people who may not understand or know how to deal with their reactions.

The factors that affected participants’ access to services can be divided into five main categories: attitudes, knowledge and understanding, sensory issues, communication, and venue accessibility. Service providers’ attitudes, knowledge, and understanding affected their ability and willingness to learn and, for instance, to provide means to communicate in writing and information in advance. Provision was improved when service providers were knowledgeable and had positive attitudes, including due to being autistic themselves or having autistic relatives. However, in other cases, they seemed to lack the understanding, for instance, that it was insufficient to express sympathy at noise disturbance and that action was required to remove the problem. Members of the public’s negative attitudes could also make it more difficult to access services, particularly if they led to bullying without intervention by the service provider to prevent this.

Venue accessibility was important and affected by factors such as location and the ability to get there without needing to use transport, which was frequently a barrier, as well as the ease of navigation around the venue. Good signage and the availability of assistance could make it easier to reach the desired point in large buildings, such as hospitals. Online location could act as an enabler, though meetings online could also be difficult in line with Zolyomi et al. [22]. Although participants did not explicitly mention this, the local availability of services would probably act as an enabler, as this would reduce the need for travel.

### 5.2. Strategies to Manage and Improve Access to Services

Participants generally seemed to have positive and pragmatic attitudes to finding workarounds and using strategies to enable them to access services. In particular, they did not express any negative feelings about themselves due to sensory and communication issues and the mismatch between their needs and approaches and those of the majority non-autistic population. Their focus was on managing their needs to remove stressors and barriers to access. It would be interesting to investigate whether this type of approach is more common for older autistic people who may have gained confidence and skills in overcoming barriers throughout their lives. However, several participants indicated that they had lost resilience and energy over time, including due to burnout.

The majority of the strategies mentioned by participants related to managing sensory issues, particularly sensory overstimulation. They included reducing or eliminating it through the use of protection, finding calm spaces, having strategies to calm themselves, asking for the first appointment, drawing attention to noise problems and asking for something to be changed, and trying to find sensorially calm services. There has been some discussion of the particular strategies used by autistic people to manage noise and other sensory issues, e.g., [19], but not all the strategies suggested here are covered. The use of noise protection and sunglasses was generally reasonably successful, as was the use of a purring furry toy and a light and cloud ceiling display as calming strategies. While the latter seems to have been used at night rather than during service use, having calming strategies to use after or before service use may have improved participants’ experiences, and the need for calming strategies could have been a consequence of service use.

The use of protection had the advantage of not requiring co-operation from other people, unlike the use of quiet spaces and requiring other people to take action to reduce noise. The use of noise protection is in line with that of noise-cancelling headphones and music, e.g., [20]. Calm spaces to retreat to could be a good solution, and their use parallels that of avoiding sensory exposure in Zheng et al. [20] and the suggestion for providing quiet waiting areas in Simpson [32]. However, access to quiet spaces depended on their availability or the ability to organise one. In addition, the success of such spaces required other people to respect them by remaining quiet. Problems in making other people respect quiet spaces are in line with the grey literature on the analogous difficulties in quiet carriages on trains, e.g., [45].

Making other people take action about noise or other sensory stimulation could also be difficult, as this was again something participants did not have direct control over. This was sometimes successful, but at others, verbal sympathy was received and not the action they were asking for. The lack of response, particularly in the healthcare system, is, unfortunately, in line with the literature on medical professionals’ lack of understanding of autism, e.g., [25,29]. A preference for the first appointment to reduce waiting parallels a suggestion in [23] for first or last appointments to remove barriers to visiting GPs.

Other strategies, such as choosing calm services and finding a quiet space to escape to, depended on their availability and the co-operation of other people to ensure they remained calm. Asking other people to take action to reduce noise had varying success, as it depended on them understanding the need and being willing and able to make changes. In general, it seemed that strategies which depended solely on (older) autistic people such as using protection or calming strategies were more likely to be successful than those which depended on other people, such as asking them to reduce noise or finding quiet spaces to escape to.

Participants used a variety of approaches to manage communication and to try to achieve what they needed to with varying degrees of success. However, communication was very much something they needed to manage, both to remove barriers to accessing services and to avoid or reduce stress. Their strategies were largely based on minimising interaction and the use of written communication, including images. Strategies to avoid interaction included trying to choose services which did not have a reception desk or which did not require interaction at the reception desk. This depended on the availability of such services.

Strategies based on written communication included asking other people to write things down to avoid non-verbal communication, a preference for text and email communication, and trying to obtain an email contact address for a named individual, for instance, at a healthcare provider. Related strategies included the use of photos, for instance, to explain the desired hairstyle to hairdressers. The preference for written communication is in line with comments in the literature on (older) autistic people’s preferences for asynchronous written communication [13,20] and text or online appointment booking for medical appointments [23]. A further strategy involved trying to obtain more time to respond to questions, for instance by finding services which allowed this. Obtaining the email address of a named individual seemed to be surprisingly difficult and time-consuming. The success of the other strategies was variable and depended on other people’s understanding and willingness to co-operate. Participants would also have liked to have a personal assistant to help with communication.

The main strategies to manage the lack of knowledge and understanding and, to some extent, also to counter negative attitudes were the provision of information that the participant was autistic and their requirements. For instance, two participants indicated that stating they were autistic and explaining their needs was successful in having them met, in one case, due to greater understanding as the service provider, in this case, their hairdresser, had an autistic son. However, this approach was not always successful, particularly in a medical context. This indicates that services providers’ responses may be determined by their attitudes and knowledge. The negative response in a medical context is, unfortunately, unsurprising in view of the literature on medical professionals’ lack of understanding of autism and need for training, e.g., [9,28,29]. Some types of service providers may also be more open to welcoming (older) autistic people and adapting their services to be (older) autistic-friendly. The factors that affect service providers’ responses and their willingness to become (older) autistic-friendly require further research.

Other strategies for managing services included using online services, particularly those that avoided human contact, as well as online spaces that had clear rules, and in-person services without staffed reception desks. The success of this approach depended on the availability and suitability of such spaces and services. However, there were also indications from participants that online participation also had its difficulties, possibly paralleling comments in Zolyomi et al. [22] on sensory issues and other problems in online meetings.

## 6. Conclusions

This paper has presented new empirical data on the experiences of older autistic people of accessing and using services. It used this data to answer two research questions: 1. the factors that affect service access and experiences; and 2. the strategies participants used to improve their access to and experiences of using services.

The results show older autistic people as autonomous adults taking action to try to overcome the barriers they experience to accessing services on the same terms as everyone else. However, overall, participants had more negative than positive experiences of using services and, despite the use of a range of strategies, did not always achieve equal access.

In line with the literature, mainly for younger autistic adults, the healthcare and public transport systems were found to be particularly problematical in both sensory and communication terms. These are both important services and these difficulties are reflected in (older) autistic people’s poor health outcomes, and their reduced ability to access other services and participate in society, respectively.

We categorised the factors that affected service access and experiences of service use into five main categories: sensory issues, communication issues, attitudes, knowledge and understanding, and venue accessibility. The lack of understanding of their sensory and communication needs and provision to meet them caused participants major avoidable stress and to spend a lot of time and energy trying to manage their needs. They used a variety of strategies to achieve this, with a high proportion of strategies related to managing sensory issues, particularly noise. This included both strategies that participants could apply independently, such as using noise protection and sunglasses, and those that required co-operation from others, such as taking action to reduce noise, providing and respecting quiet spaces and requesting the use of written communication and the provision of an email address for direct contact with a named person. In the latter case, responses were not always positive and there were sometimes extensive delays.

There were also strategies for trying to manage knowledge and understanding, such as informing service providers they were autistic and of their requirements. Success was variable and less so in medical compared to other contexts and may have depended on existing attitudes. Participants managed venue accessibility, as well as communication and sensory issues, by using services online where available and suitable.

### 6.1. Strengths and Weaknesses of This Work

The greatest strengths of this work were the co-production approach involving a team of older people supported by an advisory committee of older autistic people and the centrality of older autistic perspectives and experiences to all aspects of this work. This also shows the ability of older autistic people to work successfully together to obtain important research outcomes. A further strength was the move away from a deficit perspective, again drawing on the co-production approach, and the rich data obtained and presented.

Co-production approaches are an important way of showing respect for the community being researched, enabling this community to participate in deciding the research questions and ensuring that the research outcomes will be useful to the community. There is also considerable anecdotal evidence that many autistic people are suspicious of non-autistic researchers. Involving (older) autistic researchers is an ethical way to build trust in the community, increase participation, and make participants more open to sharing their experiences. In addition, the involvement of (older) autistic researchers provided an ‘insider’ perspective and may have resulted in a greater understanding of and insights into participants’ experiences. It also avoided any risk of trivialising or applying a deficit-based approach to the difficulties experienced by participants.

It was also the co-production approach and involvement of the older autistic community which led to the inclusion of the response option ‘A guide to autistic-friendly services’. This made possible the prioritisation of the development of a website to provide this guide through reviews by older autistic people for older autistic people. Thus, without the co-production approach, the project would have taken a totally different direction which may have been less in line with the wishes of the older autistic community.

The main limitations were the relatively small number of participants and their lack of diversity, including the lack of older autistic people from ethnic minorities, with learning disabilities, and who were over 80. While this is not untypical, we recognise the importance of covering the full diversity of the older autistic community, including from these groups. A further limitation was the fact that we did not ask for demographic data from interview and focus group participants. We were not able to extract this from the questionnaire responses due to the early anonymisation of the data.

### 6.2. Recommendations

The analysis of the data, the factors that affect access to services and experiences of using them, and participants’ strategies were used to develop the following recommendations for service providers.

Training and attitudes
  1. Provide training developed and taught by (older) autistic people on their experiences and needs to all personnel.
  2. Avoid stereotypes of (older) autistic people and listen to them.
Physical access
  3. Provide good quality online services in addition to in-person ones where feasible.
  4. Provide assistance to get to appointments in large buildings, such as hospitals.
Communication
  5. Provide options to book appointments using email and online forms.
  6. Minimise the personal interaction required to obtain access to services and avoid chatty or gatekeeping reception desks.
  7. Provide an option for information and questions to be made available in writing in advance of appointments.
  8. Provide options to communicate in writing during appointments.
  9. Allow additional time for (older) autistic people to provide information or respond to questions.
Sensory issues
  10. Provide separate calm waiting areas and calm spaces to escape to where talking is not permitted.
  11. Check in advance whether service users want music, radio, etc., off or on during appointments.
  12. Arrange a different appointment location in the event of repairs or other noisy work nearby or, if this is not possible, check whether the service user prefers the appointment to be rescheduled.

### 6.3. The Main Contributions of This Work

Several of the results simply confirm results in the existing literature. However, overall, this work has the following main contributions:

1. Categorising the factors that affect service access and experiences into five groups.
2. Using the voices of older autistic people, which shows them as autonomous adults who experience numerous difficulties in accessing services and who have a range of strategies to manage these difficulties, though not always successfully.
3. Providing details of the factors that affect older autistic people’s access to and experiences of service use, particularly in the areas of healthcare and transport.
4. Providing a wide range of strategies used by older autistic people to improve their access to and experiences of services and an evaluation of their success.
5. Providing a list of recommendations divided into categories for improving service provision for (older) autistic people.

### 6.4. Further Work

This paper has contributed to filling the knowledge gaps in this area, but there is still only very limited research on older autistic people’s experience of service use, the barriers that affect access, and the strategies used to overcome them. The following specific areas requiring further research were identified in this paper.

1. Whether participants’ pragmatic attitudes to their sensory issues and focus on managing them to remove stressors and access barriers is typical of autistic people in general and, if not, the factors that affect this and whether they include age.
2. Whether participants’ positive and pragmatic attitudes to finding workarounds and using strategies to enable them to access services is more common for older autistic people and, if so, whether this is due to them having gained confidence and skills in overcoming barriers throughout their lives.
3. A detailed investigation of the ways in which ageing affects sensory issues for autistic people and the factors that affect this.
4. The factors that affect service providers’ responses and their willingness to become (older) autistic-friendly.
5. The extent to which service providers’ reactions to disclosure that the participant was autistic and their requirements depended on the type of service provider or positive existing attitudes and the lack of stereotypical views about what being autistic involved.
6. Investigating the experiences of a more diverse group of older autistic people including from ethnic minorities and with learning disabilities. In the latter case, appropriate approaches to involving them and obtaining information will be required, and this may require assistance from support people. However, the focus should be on the older autistic people and the presence of support people to assist them and help them present their views using whatever communication strategies they find most appropriate.
7. Investigating the views of support people, including family members, friends, and carers, including any concerns they have as to what will happen if and when they are no longer able to provide support. This should be considered complementary to investigating the experiences and views of older autistic people.

## Data Availability

Anonymised data, for which the participants gave permission, have been deposited in the University of Glasgow data repository. They can be accessed at http://doi.org/10.5525/gla.researchdata.1386. However, this only includes the questionnaire data and not the focus group and interview transcripts, which are more difficult to anonymise.

## Appendix A. Focus Group and Interview Questions

### Appendix A.1. Autistic-Friendly Spaces and Services

1. Can you provide any examples of spaces or services you find autistic-friendly, at least to some extent? Are there any other types of autistic-friendly spaces and services you would be interested in?
2. What makes a space or service autistic-friendly? Are there any differences in autistic-friendly online and real spaces and services?
3. What types of information do you want about these spaces and services?
4. Are you interested in information specially tailored to meet your needs and interests? If yes, how would you like this to be provided? Do you have any privacy concerns if you provide personal information to support this?
5. Do you already use any websites or apps to find autistic-friendly spaces or services? If so, what do you like and not like about them? What other websites or apps, if any, are you aware of to support finding autistic-friendly spaces or services? If you are, what do you like and not like about them?
6. Do you have any suggestions for what to call this set of features and functions?

### Appendix A.2. Organisation, Executive Functioning, and Life Tips

Many autistic (and non-autistic) people have difficulties with organising, planning, getting round to doing things, prioritising, and decision making. This may include difficulties with housework, shopping, form filling, or managing time. We want to know how an app or website could help support older autistics with these activities. The aim of these questions is to help us develop a website or app which would provide this type of support. We are asking about the content, not its accessibility features.

1. What types of issues/activities related to the above might you find it useful to have support with?
2. What type of support would you find useful? (Do not worry whether systems to provide this type of support are currently available.)
3. Do you want a general version for all users or a version that can be tailored to you? Do you have any privacy concerns if you provide personal information to support this?
4. Do you already use any websites or apps to help with organising your life, prioritising things, decision making, etc.? If so, what do you like and not like about them?

(Prompt: e.g.,: https://habitica.com/, acessed on 12 November 2024)

What other websites or apps, if any, are you aware of to help with organising your life, prioritising things, decision making, etc.? If any, what do you like and not like about them?

5. Do you have any suggestions for what to call this set of features and functions?

## References

[1] Eurostat (2023). Population on 1 January by Age Group and Sex. https://ec.europa.eu/eurostat/databrowser/view/DEMO_PJANGROUP__custom_1269323/bookmark/table?lang=en&bookmarkId=66c88f91-bde0-4606-abbd-bd1a65c8224a.

[2] Sacco R., Camilleri N., Eberhardt J., Umla-Runge K., Newbury-Birch D., Carotenuto M. (2022). The prevalence of autism spectrum disorder in Europe. Autism Spectrum Disorders—Recent Advances and New Perspectives.

[3] Davids R.C., Groen Y., Berg I.J., Tucha O., van Balkom I.D. (2020). Local-global processing approaches in older autistic adults: A matched control study using RCFT and WAIS-IV. Res. Autism Spectr. Disord..

[4] Tse V.W., Crabtree J., Islam S., Stott J. (2019). Comparing intellectual and memory abilities of older autistic adults with typically developing older adults using WAIS-IV and WMS-IV. J. Autism Dev. Disord..

[5] Sonido M., Arnold S., Higgins J., Hwang Y.I.J. (2020). Autism in later life: What is known and what is needed?. Curr. Dev. Disord. Rep..

[6] Geurts H.M., McQuaid G.A., Begeer S., Wallace G.L. (2022). Self-reported parkinsonism features in older autistic adults: A descriptive study. Autism.

[7] Hand B.N., Angell A.M., Harris L., Carpenter L.A. (2019). Prevalence of physical and mental health conditions in Medicare-enrolled, autistic older adults. Autism.

[8] Nylander L., Axmon A., Björne P., Ahlström G., Gillberg C. (2018). Older adults with autism spectrum disorders in Sweden: A register study of diagnoses, psychiatric care utilization and psychotropic medication of 601 individuals. J. Autism Dev. Disord..

[9] Mason D., Ingham B., Urbanowicz A., Michael C., Birtles H., Woodbury-Smith M., Brown T., James I., Scarlett C., Nicolaidis C. (2019). A systematic review of what barriers and facilitators prevent and enable physical healthcare services access for autistic adults. J. Autism Dev. Disord..

[10] Tse V.W., Lei J., Crabtree J., Mandy W., Stott J. (2022). Characteristics of older autistic adults: A systematic review of literature. Rev. J. Autism Dev. Disord..

[11] Office of National Statistics (2020). Office of National Statistics Outcomes for Disabled People in the UK. https://www.ons.gov.uk/peoplepopulationandcommunity/healthandsocialcare/disability/articles/outcomesfordisabledpeopleintheuk/2020.

[12] Paradiz V. (2009). Autism, Identity and Employment, Transcending the Obstacles of the Working World. Autism Advocate, 1st ed. https://www.rwjbh.org/documents/csh/kohls/autism-identity-employment.pdf.

[13] Hersh M., Elley S., Banks Z., Mery P., Dawson M., Cowan D., Watson C., Milton D., Ridout S., Martin N., Mills R., Murray D. (2020). Accessing services and social interaction: Strategies used by autistic people. Neurodiversity Reader.

[14] Dinishak J. (2022). The deficit view and its critics. Disabil. Stud. Q..

[15] PARC (Undated) Participatory Autism Research Collective. https://participatoryautismresearch.wordpress.com/.

[16] Milton D., Ridout S., Martin N., Mills R., Murray D. (2020). Neurodiversity Reader.

[17] AASPIRE (2020). Academic Autism Partnership in Research and Education. https://aaspire.org/.

[18] Arnold S.R., Higgins J.M., Weise J., Desai A., Pellicano E., Trollor J.N. (2023). Towards the measurement of autistic burnout. Autism.

[19] MacLennan K., Woolley C., Emily, Heasman B., Starns J., George B., Manning C. (2023). “It is a big spider web of things”: Sensory experiences of autistic adults in public spaces. Autism Adulthood.

[20] Zheng L., Foley K.-R., Grove R., Elley K., Brown S.A., Leong D.-J., Li X., Pellicano E., Trollor J.N., Hwang Y.I. (2022). The use of everyday and assistive technology in the lives of older autistic adults. Autism.

[21] Howard P.L., Sedgewick F. (2021). ‘Anything but the phone!’: Communication mode preferences in the autism community. Autism.

[22] Zolyomi A., Begel A., Waldern J.F., Tang J., Barnett M., Cutrell E., McDuff D., Andrist S., Morris M.R. (2019). Managing stress: The needs of autistic adults in video calling. Proc. ACM Hum.-Comput. Interact..

[23] Doherty M., Neilson S., O’Sullivan J., Carravallah L., Johnson M., Cullen W., Shaw S.C. (2022). Barriers to healthcare and self-reported adverse outcomes for autistic adults: A cross-sectional study. BMJ Open.

[24] Nicolaidis C., Raymaker D., McDonald K., Dern S., Boisclair W.C., Ashkenazy E., Baggs A. (2013). Comparison of healthcare experiences in autistic and non-autistic adults: A cross-sectional online survey facilitated by an academic-community partnership. J. Gen. Intern. Med..

[25] Westminster Commission on Autism (2016). A Spectrum of Obstacles: An Inquiry into Access to Healthcare for Autistic People. https://westminsterautismcommission.files.wordpress.com/2016/03/ar1011_ncg-autism-report-july-2016.pdf.

[26] Nicolaidis C., Raymaker D.M., Ashkenazy E., McDonald K.E., Dern S., Baggs A.E., Kapp S.K., Weiner M., Boisclair W.C. (2015). “Respect the way I need to communicate with you”: Healthcare experiences of adults on the autism spectrum. Autism.

[27] Wilson S. (2017). How Is Counselling Experienced by People with Asperger’s Syndrome?: A Qualitative Study. Doctoral Dissertation.

[28] Nicolaidis C., Schnider G., Lee J., Raymaker D.M., Kapp S.K., A Croen L., Urbanowicz A., Maslak J. (2021). Development and psychometric testing of the AASPIRE adult autism healthcare provider self-efficacy scale. Autism.

[29] Unigwe S., Buckley C., Crane L., Kenny L., Remington A., Pellicano E. (2017). GPs’ confidence in caring for their patients on the autism spectrum: An online self-report study. Br. J. Gen. Pract..

[30] Jones L., Goddard L., Hill E.L., Henry L.A., Crane L. (2014). Experiences of receiving a diagnosis of autism spectrum disorder: A survey of adults in the United Kingdom. J. Autism Dev. Disord..

[31] Bradshaw P., Pellicano E., van Driel M., Urbanowicz A. (2019). How can we support the healthcare needs of autistic adults without intellectual disability?. Curr. Dev. Disord. Rep..

[32] Simpson S. (2020). Creating accessible healthcare environments for people with autism. Nurs. Times.

[33] Nicolaidis C., Raymaker D., McDonald K., Kapp S., Weiner M., Ashkenazy E., Gerrity M., Kripke C., Platt L., Baggs A. (2016). The development and evaluation of an online healthcare toolkit for autistic adults and their primary care providers. J. Gen. Intern. Med..

[34] Wilson N.J., Stevens A., Srasuebkul P., Kersten M., Lin Z., Trollor J.N., Arnold S.R. (2021). Exploring the relationship between community mobility and quality of life, employment and completing further education for autistic adults. J. Transp. Health.

[35] Deka D., Feeley C., Lubin A. (2016). Travel patterns, needs, and barriers of adults with autism spectrum disorder: Report from a survey. Transp. Res. Rec..

[36] Lubin A., Feeley C. (2016). Transportation issues of adults on the autism spectrum: Findings from focus group discussions. Transp. Res. Rec..

[37] Pfeiffer B., Song W., Davidson A., Salzer M., Feeley C., Shea L. (2024). Transportation Use and Barriers for Employed and Unemployed Autistic Adults. Autism Adulthood.

[38] Joffe H., Yardley L., Marks D., Yardley L. (2003). Content and thematic analysis. Research Methods for Clinical and Health Psychology.

[39] Office for National Statistics (ONS) Released 3 April 2023, ONS Website, Article, Profile of the Older Population Living in England and Wales in 2021 and Changes Since 2011. https://www.ons.gov.uk/peoplepopulationandcommunity/birthsdeathsandmarriages/ageing/articles/profileoftheolderpopulationlivinginenglandandwalesin2021andchangessince2011/2023-04-03.

[40] APA, American Psychiatric Association, D.S.M.T.F., DSM-5 Task Force (2013). Diagnostic and Statistical Manual of Mental Disorders: DSM-5 (Vol. 5, No. 5).

[41] Elwin M., Schröder A., Ek L., Wallsten T., Kjellin L. (2017). Sensory clusters of adults with and without autism spectrum conditions. J. Autism Dev. Disord..

[42] Charlton R.A., McQuaid G.A., Wallace G.L. (2024). Exploring the effects of age and sex on sensory sensitivities in middle and older aged autistic adults. Res. Autism Spectr. Disord..

[43] Morgan H. (2019). Connections Between Sensory Sensitivities in Autism; the Importance of Sensory Friendly Environments for Accessibility and Increased Quality of Life for the Neurodivergent Autistic Minority. PSU McNair Sch. Online J..

[44] Weiss J.A., Fardella M.A. (2018). Victimization and perpetration experiences of adults with autism. Front. Psychiatry.

[45] Anon (Undated) How Often Are Quiet Coaches Actually Quiet. https://www.railforums.co.uk/threads/how-often-are-quiet-coaches-actually-quiet.245317/.

